# Second Medical School in Louisville

**Published:** 1848-03

**Authors:** 


### SECOND MEDICAL SCHOOL IN LOUISVILLE.

The attempt to establish a second medical school in this city, (announced in our last number) seems for the present to be a failure. The bill for a charter, was lost in the Senate, by a vote of 18 to 10. The application was then transferred to the lower House, and referred to the Committee on Education, who reported the same bill; but up to this date (February 26th) the report has not been called up; and the Legislature, by a joint resolution, is to adjourn on the 28th; we may presume that the friends of the measure will not make any further attempts to procure its passage at the present session. This postponement will afford time for the people of Louisville and of the State of Kentucky, to ponder on the question, whether, if four hundred passengers wished to ascend the Falls, it would be better to divide them between two steamboats of feeble power, or keep them in one, of such size and strength, that it could stem the current, as successfully with the whole as a part. They who think, that, referring equally to the interests of society in the west, and to the prosperity of the people of Louisville, a second school ought not to be established, *at this time*, have nothing to fear, and much to expect from a full and scrutinizing investigation, and such an one, we hope, will be made.

